# Supplementary material for: Chemical Constituents and Molecular Mechanism of the Yellow Phenotype of Yellow Mushroom (Floccularia luteovirens)
Source: J Fungi (Basel). 2022 Mar 18;8(3):314. doi: 10.3390/jof8030314 (PMC8949800; doi:10.3390/jof8030314)
Supplement: Supplementary file 1 [file jof-08-00314-s001.zip › Figure S1.pdf]

A

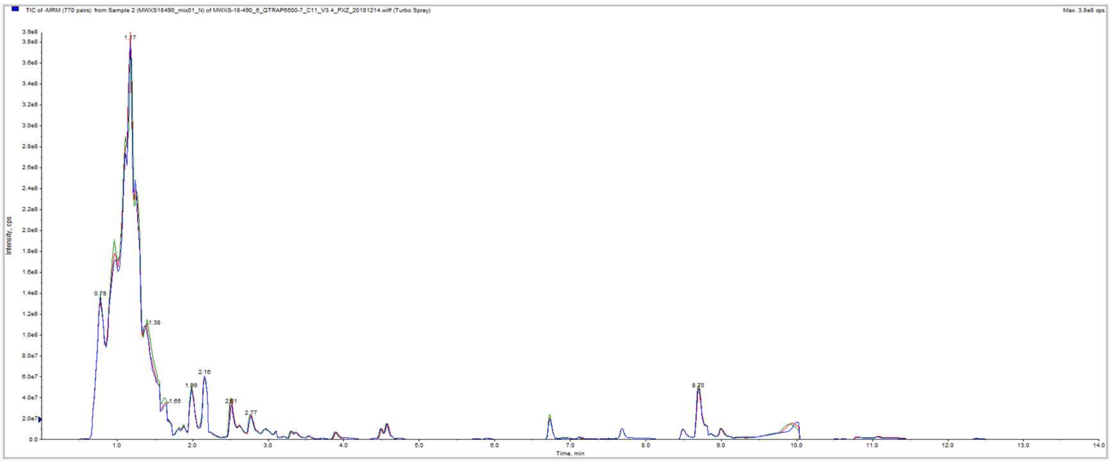

B

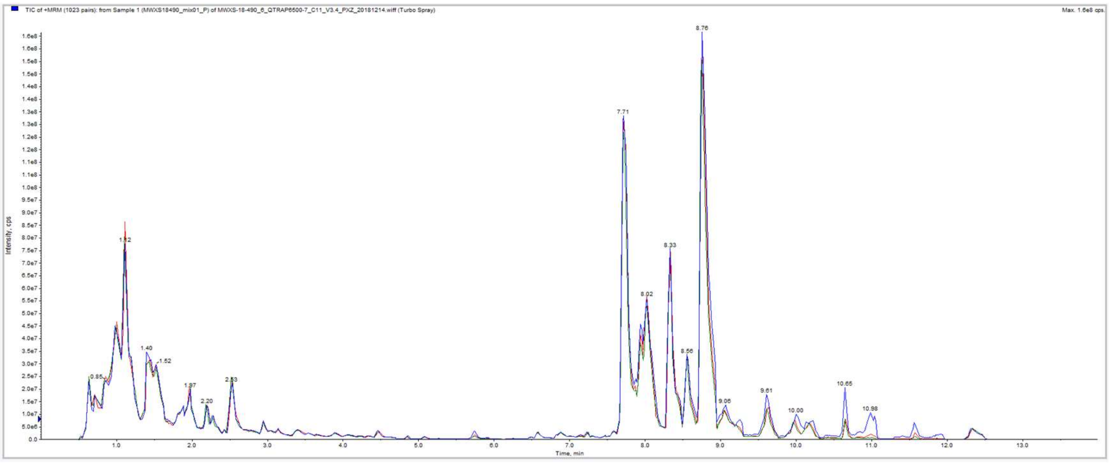

Figure S1. The TIC overlaps by QC sample Mass Spectrometry. The pictures of (**A**) white sample (**B**) yellow sample shows that the curves of total ion current detected by metabolite were highly overlapping. The retention time and peak intensity were consistent, indicating that the signal stability number of the same sample is detected by mass spectrometry at different time. The high stability of the instrument provides an important guarantee for the repeatability and reliability of the data.
