# Supplementary material for: Chemical Constituents and Molecular Mechanism of the Yellow Phenotype of Yellow Mushroom (Floccularia luteovirens)
Source: J Fungi (Basel). 2022 Mar 18;8(3):314. doi: 10.3390/jof8030314 (PMC8949800; doi:10.3390/jof8030314)
Supplement: Supplementary file 1 [file jof-08-00314-s001.zip › Tables S3-S6.pdf]

**Table S3.** Distribution of the Clean Reads of the sequencing.

| Sample | Total Raw Reads(Mb) | Total Clean Reads(Mb) | Total Clean Bases(Gb) | Clean Reads Q20(%) | Clean Reads Q30(%) | Clean Reads Ratio(%) |
|---|---|---|---|---|---|---|
| White | 52.66 | 52.65 | 7.9 | 97.19 | 92.89 | 99.99 |
| Yellow | 50.29 | 50.29 | 7.54 | 97.64 | 93.96 | 99.99 |

**Table S4.** Distribution of the lengths of the assembled unigenes.

| Sample | Total Number | Total Length | Mean Length | N50 | N70 | N90 | GC(%) |
|---|---|---|---|---|---|---|---|
| White | 22823 | 57646679 | 2525 | 3805 | 2626 | 1353 | 45.33 |
| Yellow | 21023 | 47993042 | 2282 | 3506 | 2357 | 1218 | 45.45 |
| All-Unigene | 43750 | 1.06E+08 | 2424 | 3782 | 2529 | 1298 | 46.53 |

**Table S5.** Phylogenetic analysis of KOG species.

| species | gene numbers | percentage |
|---|---|---|
| Agaricus bisporus var. bisporus H97 | 7274 | 20.32 |
| Agaricus bisporus var. burnettii JB137-S8 | 6175 | 17.25 |
| Galerina marginata CBS 339.88 | 4136 | 11.55 |
| Laccaria amethystina LaAM-08-1 | 3885 | 10.85 |
| Laccaria bicolor S238N-H82 | 2379 | 6.65 |
| Hebeloma cylindrosporum h7 | 2019 | 5.64 |
| Leucoagaricus sp. SymC.cos | 1776 | 4.96 |
| Hypholoma sublateritium FD-334 SS-4 | 1037 | 2.9 |
| others | 7085 | 19.88 |

**Table S6.** The primers used in this study.

| Primer name | Sequence |
|---|---|
| FlMCH5-F | ATGGAACCCTCGATACACGA |
| FlMCH5-R | TCACACTATCTGGGTCCCTC |
| FlMCH5-GUS-F | ATGGAACCCTCGATACACGA |
| FlMCH5-GUS-R | CACTATCTGGGTCCCTC |
| FlMCH5-GUS attb1 | AAAAAGCAGGCTTCATGGAACCCTCGATACACGATG |
| FlMCH5-GUS attb2 | AGAAAGCTGGGTCCACTATCTGGGTCCCTCTTT |
| FlMCH5-XbaI | ggtctagaATGGAACCCTCGATACACGA |
| FlMCH5-BamHI | ggggatccTCACACTATCTGGGTCCCTC |
