# Supplementary figures and images for: Chemical Constituents and Molecular Mechanism of the Yellow Phenotype of Yellow Mushroom (Floccularia luteovirens)
Source: J Fungi (Basel). 2022 Mar 18;8(3):314. doi: 10.3390/jof8030314 (PMC8949800; doi:10.3390/jof8030314)

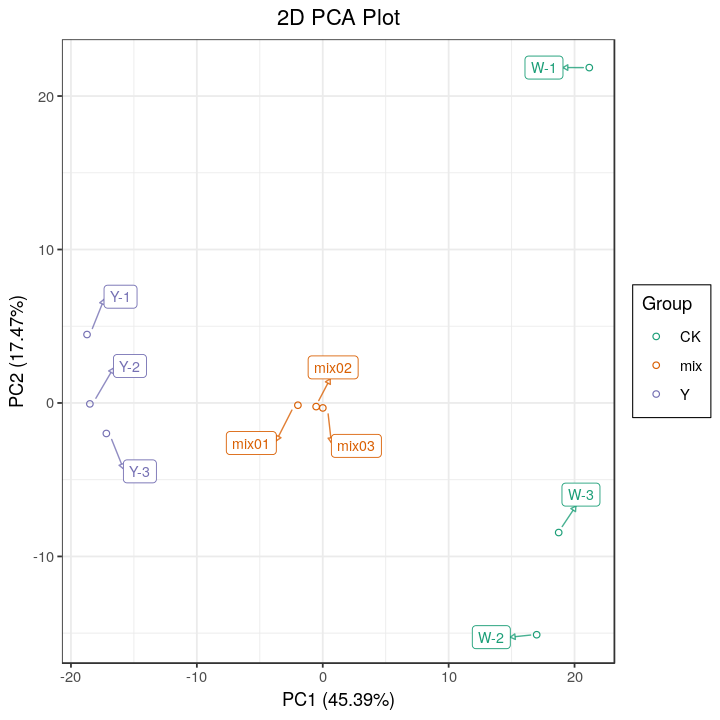

Supplement: Supplementary file 1 [file jof-08-00314-s001.zip › Figure S2.png]

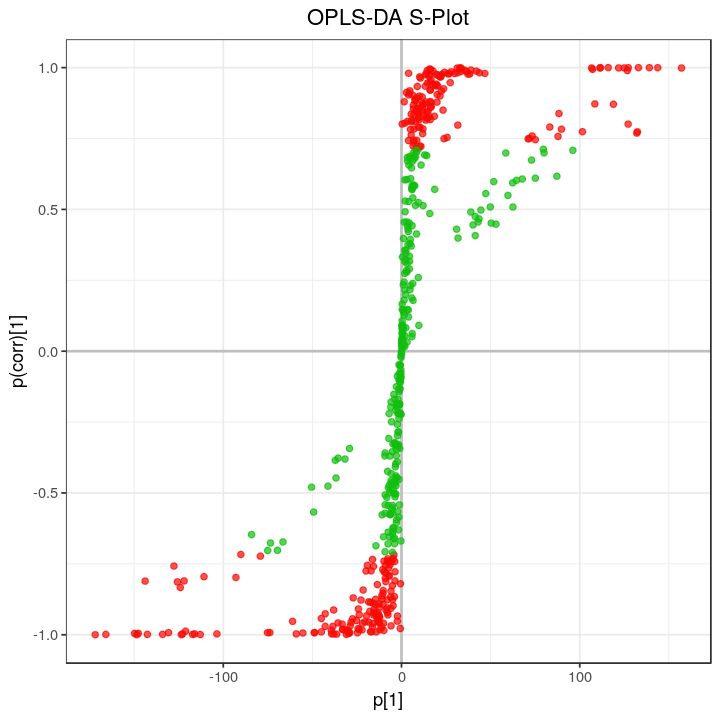

Supplement: Supplementary file 1 [file jof-08-00314-s001.zip › Figure S3.png]

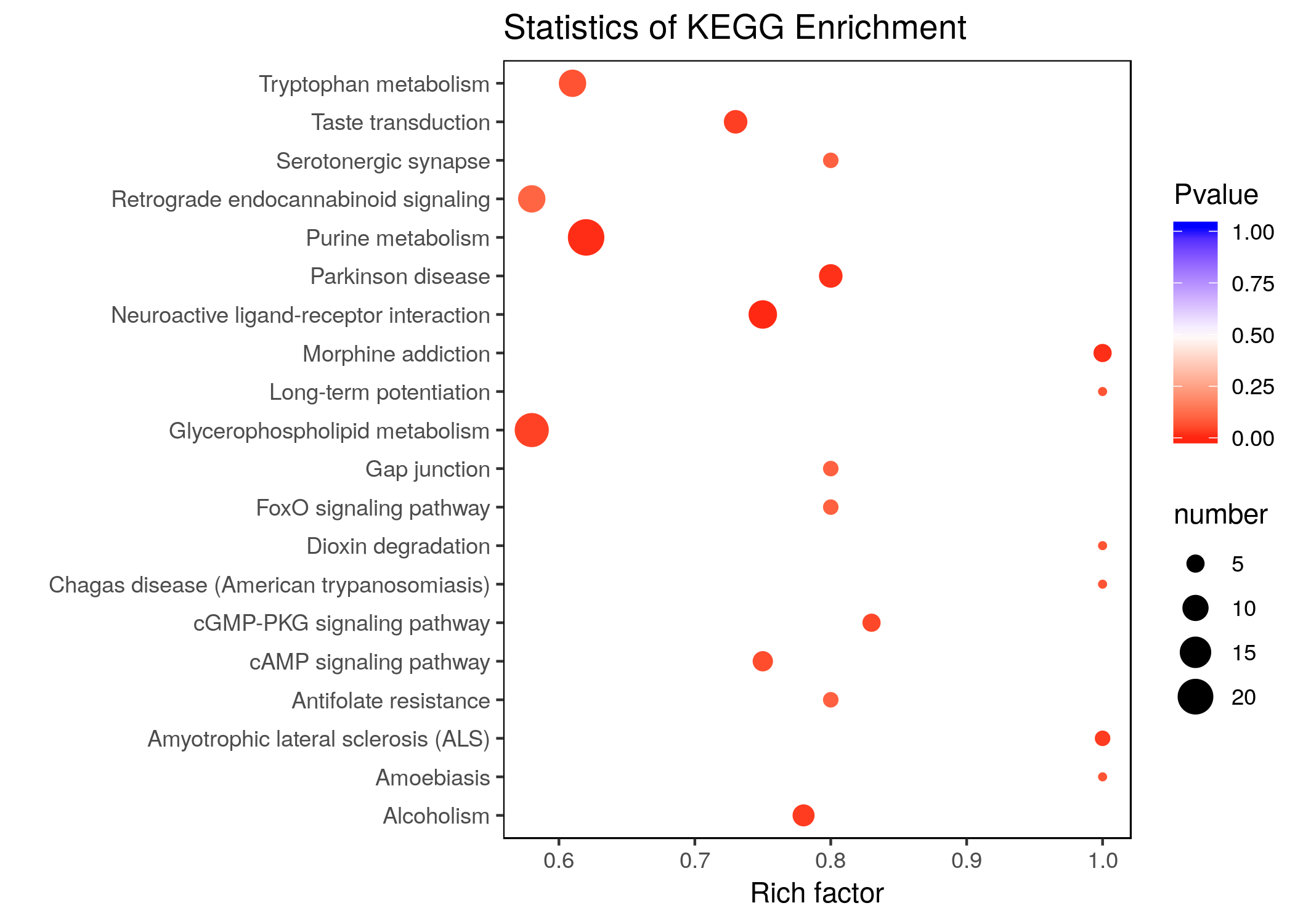

Supplement: Supplementary file 1 [file jof-08-00314-s001.zip › Figure S4.png]

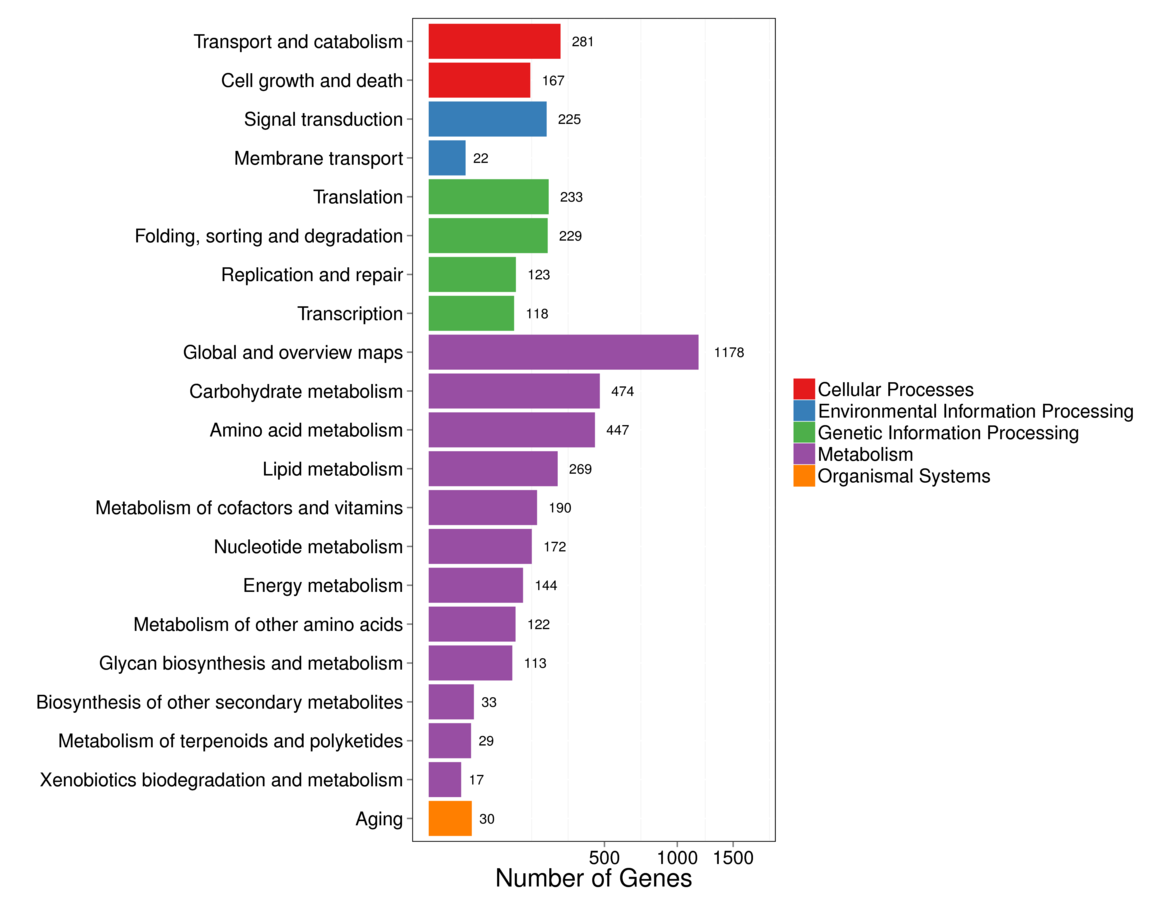

Supplement: Supplementary file 1 [file jof-08-00314-s001.zip › Figure S5.png]

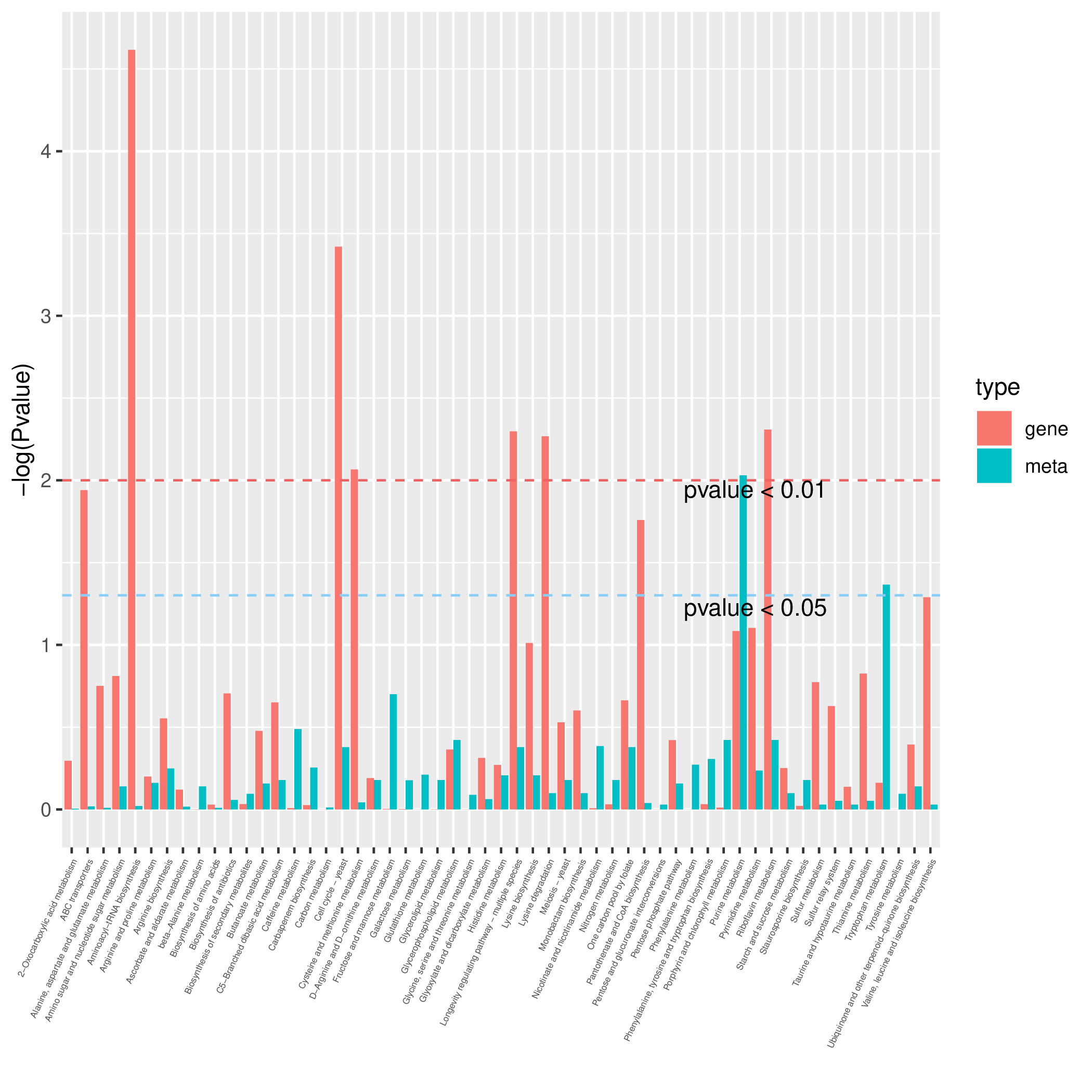

Supplement: Supplementary file 1 [file jof-08-00314-s001.zip › Figure S6.png]
